# The microbiotest battery as an important component in the assessment of snowmelt toxicity in urban watercourses—preliminary studies

**DOI:** 10.1007/s10661-014-4252-1

**Published:** 2015-01-28

**Authors:** S. Szklarek, M. Stolarska, I. Wagner, J. Mankiewicz-Boczek

**Affiliations:** 1 European Regional Centre for Ecohydrology, Polish Academy of Sciences, 3, Tylna Str., 90-364 Lodz, Poland; 2 Department of Applied Ecology, University of Lodz, ul Banacha 12/16, 90-237 Lodz, Poland; 3 WIND-HYDRO, Opiekuncza 19 Str, 90-411 Lodz, Poland

**Keywords:** Toxicity, Microbiotest, Urban runoff, Storm water, Snowmelt

## Abstract

The aim of the study was to use a battery of biotests composed of producers (*Selenastrum capricornutum*, *Sorghum saccharatum*, *Lepidium sativum*, and *Sinapis alba*), consumers (*Thamnocephalus platyurus*), and decomposers (*Tetrahymena thermophila*) to evaluate the toxicity of snowmelt and winter storm water samples. The toxicity of the samples collected in the winter period December to February (2010–2011), in one of the largest agglomerations in Poland, the city of Lodz, was compared to that of storm water samples taken under similar conditions in June. The most toxic snowmelt samples were found to be high acute hazard (class IV), while the remaining samples were rated as slight acute hazard (class II). *L. sativum* (in the Phytotox test) was the most sensitive test organism, giving 27 % of all toxic responses, followed by *S. capricornutum* with 23 % of all responses. *T. thermophila* was the least sensitive, with only 2 % of all toxic responses. The greatest range of toxicity was demonstrated by samples from the single family house catchment: no acute hazard (class I) to high acute hazard (class IV).

## Introduction

Urban areas are hot spots that drive environmental change at multiple scales (Grimm et al. 2008) and so represent a major potential threat to the quality of aquatic ecosystems (Ballantine and Davies-Colley 2014). An important source of waterway pollution is surface runoff from urbanized areas. Although rainwater is relatively clean, Beysens et al. (2006) report that the concentration of all measured ions (H^+^, Na^+^, K^+^, Ca^2+^, Mg^2+^, Zn^2+^, Cu^2+^, Cl^−^, SO_4_^2−^, NO_3_^−^, NO_2_^−^, OH^−^) in rainwater in Bordeaux, France, mg L^−1^, was within the limits defined by the WHO for potable water (WHO 2011); it can become highly contaminated sewage after washing out accumulated pollutants from streets, sidewalks, and roofs (Göbel et al. 2007).

One of the main water pollution indicators is the amount of total suspended solids (TSS). Many studies have shown that TSS particles absorb other pollutants such as heavy metals, polychlorinated biphenyls (PCBs), polycyclic aromatic hydrocarbons (PAHs) (Gromaire-Mertz et al. 1999; Jartun et al. 2008), and nutrients (Berretta and Sansalone 2011; Lee et al. 2011) which influence the quality of the aquatic ecosystem and human health. Most of these pollutants are listed as priority substances in the Water Framework Directive of the European Commission (WFD; 2000/60/EC). Moreover, Zgheib et al. (2008, 2012) propose expanding the list of WFD priority substances to include other contaminants characteristic of urban areas, such as zinc and copper, PAHs, and pesticides, termed *urban priority substances*, which have been detected in the outlets of storm water sewage systems in the city of Paris and its suburbs.

Besides rainwater, in ecosystems of cold and temperate climates, snowmelt is also an important source of water and an important carrier of pollutants to urban watercourses. Air quality in winter is often worse than that in other seasons because of coal combustion. Due to its long duration, and hence exposure to pollution, snow absorbs more atmospheric pollutants than rain (Zhu et al. 2012). In addition, the use of deicing salt increases the levels of sodium and chloride in urban runoff (Kelting et al. 2012; Swan and DePalma 2012), which leads to physical and ecological changes in water ecosystems (Ramakrishna and Viraraghavan 2005). Furthermore, long-term accumulation of snowfall leads to the accumulation of high levels of the pollutants mentioned above, including lead, hydrocarbons, chlorides, and PCBs, as well as other metals and solids (Marsalek et al. 2000; Waara and Färm 2008; Bartlett et al. 2012b; Kelting et al. 2012; Porter-Goff et al. 2013).

Polish legislation only regulates the introduction of rainwater and meltwater to open or closed leakproof sewer systems discharged into water and soil on the basis of two indicators: TSS and petroleum hydrocarbons (Dz.U.^2009^.27.169.). The other pollutants mentioned above are not listed, despite potentially being encountered in urban storm water or snowmelt runoff. However, although more complete information about the pollutants and the potential threat to the aquatic ecosystems and to humans would be provided by extending the list of chemical indicators, their assessment is costly and time consuming in everyday practice and still does not allow the biological activity (toxicity) of possible contaminants to be determined. In this situation, an assessment of overall toxicity would be an important supplement to the requirements for the disposal of storm water and snowmelt into surface water or soil. The use of biotests incorporating organisms of different trophic levels is recommended in studies of freshwater ecosystems to determine the biological activity, i.e., the toxicity, of selected contaminants or to determine the total level of contamination (Brack et al. 2005; Mankiewicz-Boczek et al. 2008; Tuikka et al. 2011).

In urban water studies, biotests with selected organisms are most commonly used to assess the toxicity of water from storm water ponds (review by Tixier et al. 2011). Although a few publications have previously evaluated the toxicity of urban storm water or snowmelt runoff, the research tended to address single sampling stations or test organisms (Waara and Färm 2008; Bartlett et al. 2012a, 2012b; Tixier et al. 2012; Chong et al. 2013; Porter-Goff et al. 2013).

A literature review reveals growing interest in assessing the toxic influence of urban runoff on aquatic ecosystems. However, future challenges concern performing a comprehensive evaluation of toxicity using a group of organisms representing different trophic levels, comparing rainwater and snowmelt runoffs with regard to their toxic impact and spatial differentiation in identical aquatic systems, and determining the impact of different types of urban land use on toxicity.

In response to these challenges, the aim of the present study is to evaluate the potential of using a battery of biotests with acute and chronic effects, incorporating organisms of different trophic levels, to assess the toxicity of snowmelt and winter storm water runoff drained from urban areas with different dominant land use types: industrial, development of blocks of flats, and single family houses. The elaboration of efficient management strategies requires both accurate identification of the threat, including hot-spot areas and their effect on ecological and human systems, as well as an elaboration of the opportunities for urban landscape rehabilitation (the Ecohydrology concept: Zalewski 2000, 2010; Urban Ecohydrology: Wagner and Breil 2013). The paper may also be a step towards developing the Polish standards regarding the introduction of storm water and meltwater to surface water and soil, to include an indication of the real threat they pose as pollutants to aquatic ecosystems.

## Materials and methods

### Study site

**Fig. 1:**
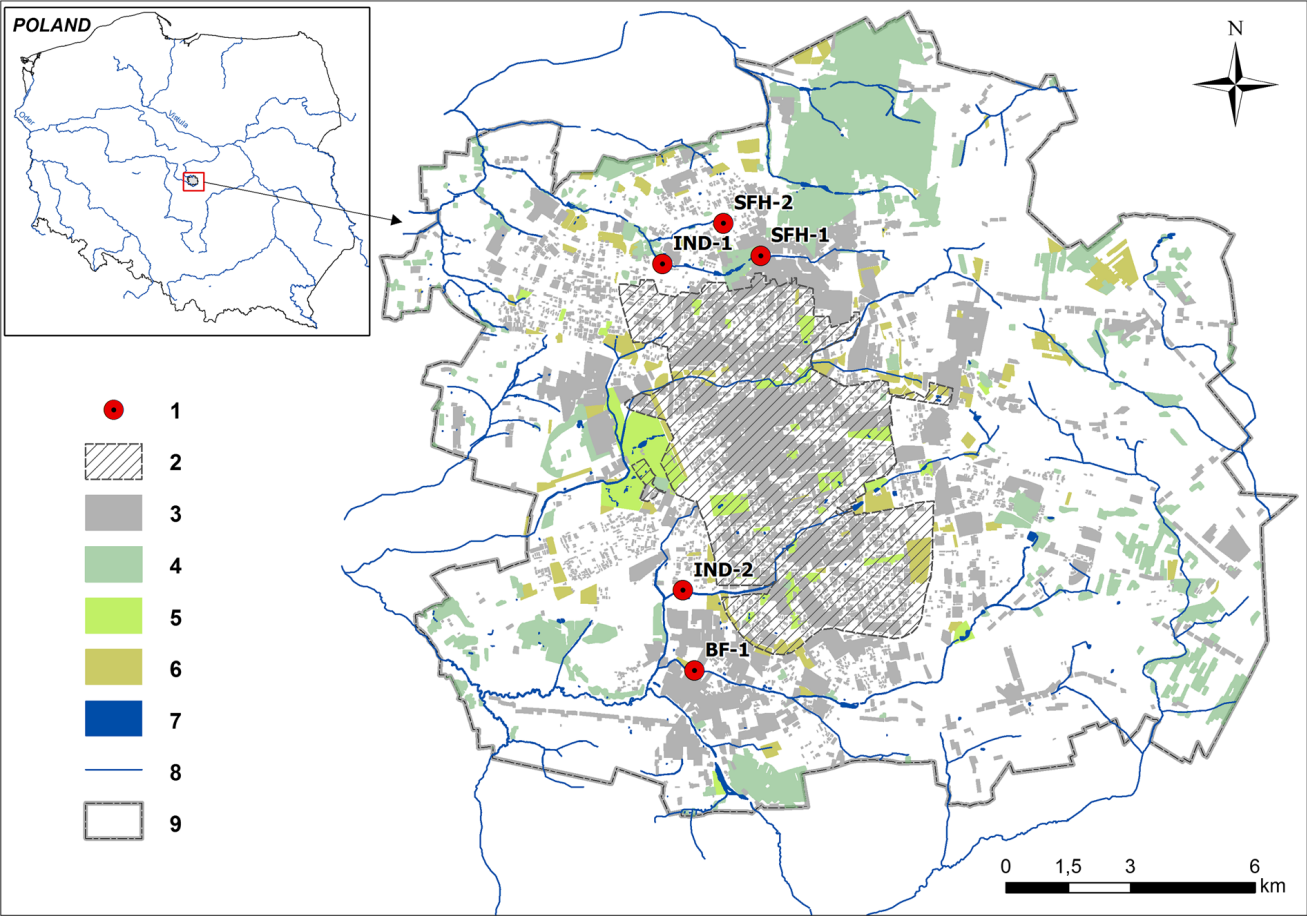
The locations of the measurement points and the key characteristics of Lodz city infrastructure and development zoning. *1* measurement points; *2* extent of combined sewage system; *3* build-up areas; *4* forest areas; *5* green spaces (parks); *6* garden plots; *7* water reservoirs; *8* river network; *9* administrative borders of the city

The city of Lodz has the third largest population index of urban centers in Poland, with 728.9 thousand inhabitants and an area of 293.3 km^2^ (GUS 2012). The city is located on the main watershed of the Oder and Vistula. The study was conducted at five outlets of the separated storm water sewer system discharging directly to four of the rivers of Lodz (Fig. 1). The five measurement points were located at outlets of storm water catchments which were chosen to represent different extents of drained area and land development types. Sampling points at outlets IND-1 and IND-2 correspond to predominantly industrial areas. Storm water catchments SNF-1 and SNF-2 are located in areas with single family houses, while BF-1 represents an area with blocks of flats as the predominant form of land development.

### Sample collection

**Fig. 2:**
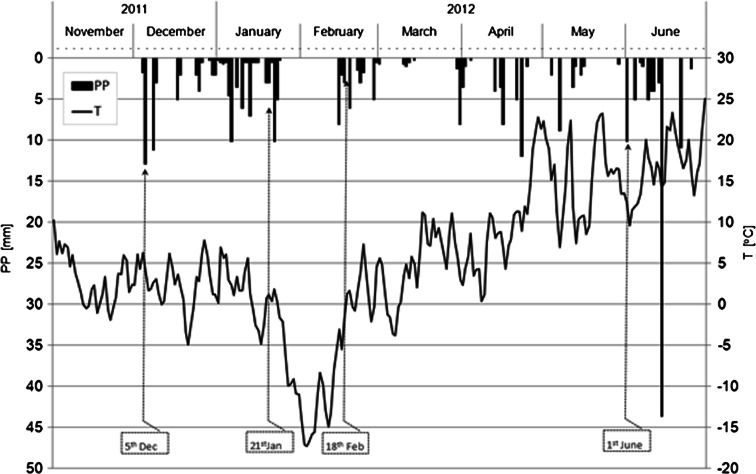
Precipitation and air temperature conditions over the study period. *PP* sum of daily precipitation and *T* water temperature; the date of sample collection is given in the *boxes*

Snowmelt and winter rain water was collected from five measurement points on the outlets of the storm water sewage system during three sampling periods (Fig. 2). Water samples from the first period (5th December 2011) came from winter rain, with a daily total of 13 mm, after 43 days of a meteorologically dry period. The second set of snowmelt water samples, taken during January 2012, was obtained from a total capacity of melting snow of approximately 47-mm cover. The third set of water samples, taken in February 2012, represents the snowmelt derived from 3-day fresh snow cover with a depth of 13.2 mm. As an additional point of reference for the toxic effect and level of contamination during winter, water samples were also taken from a late spring rainfall event: the rainfall capacity was 10.2 mm and it occurred after two dry weather weeks. Samples from point SFH-1 were taken only during the third (February) and late spring terms. All samples were collected manually at the beginning of snowmelt or rain period, when the probability of the first flash effect was the highest.

### Physicochemical analysis

All samples were analyzed in situ for pH, conductivity, dissolved oxygen, and temperature with YSI Professional Plus®. Total nitrogen (TN) was measured using persulfate digestion (Hach 1997). Total phosphorus (TP) was analyzed using the ascorbic acid method (Golterman et al. 1978). The amount of total suspended solids (TSS) was measured by gravimetry: sediments from the water samples were filtered through GF/C filters, dried at 105 °C for an hour and weighed.

The concentration of selected ions (Cl^−^, NO_2_^−^, NO_3_^−^, PO_4_^3−^, SO_4_^2−^, Na^+^, NH_4_^+^, K^+^, Mg^2+^, Ca^2+^) was analyzed using a Dionex® ion chromatograph with a cation column (CG18, IonPac CS18, CSRS-ULTRA II) and an anion column (AG22, IonPac AS22, ASRS ULTRA II). The systems were operated in isocratic elution at 30 °C at a flow rate of 1 mL min^−1^. For ion identification, combined standards were used (Dionex Corporation).

### Toxicity testing

Toxicity assessment of snowmelt and rain water samples was performed using a battery of biotests (producer MicroBioTests Inc., Belgium). Acute and chronic toxicity tests used in the study incorporated five species from different trophic levels (Table 1). The selected species (name of organisms were given according to the description in producer’s instruction) represented fresh water (*Selenastrum capricornutum*, *Thamnocephalus platyurus*, *Tetrahymena thermophila*) and soil (*Sorghum saccharatum*, *Lepidium sativum*, *Sinapis alba*) environments. The endpoints and time durations of each test were used in accordance with the producer’s instructions (Table 1).

**Table 1:** Description of the microbiotest battery applied for toxicity assessment of storm water sewage outputs

Trophic level	Organism^a^	Test name	Endpoint	Test duration (h)	Type of test	References
Producers	*Selenastrum capricornutum*	Algaltoxkit F™	Growth inhibition	72	Chronic	Algaltoxkit, 1996
	*Sorghum saccharatum*	Phytotoxkit ™	Growth inhibition	72	Chronic	Phytotoxkit, 2004
	*Lepidium sativum*					
	*Sinapis alba*					
Consumers	*Thamnocephalus platyurus*	Thamnotoxkit F™	Mortality	24	Acute	Thamnotoxkit, 1995
Decomposers	*Tetrahymena thermophila*	Protoxkit F™	Growth inhibition	24	Acute	Protoxkit, 1998

^a^Organism name is given according to the description in producer’s instruction (MicroBioTests Inc., Belgium)

The response of the organism was classified as toxic when the percentage effect (PE) of mortality was equal to or higher than 10 % and growth inhibition was equal to or higher than 20 % according to previous studies (Kaza et al. 2007; Mankiewicz-Boczek et al. 2008). Acute hazard classes of samples were assessed according to Persoone et al. (2003).

## Results

### Physicochemical analysis

The highest temperature of water samples in winter was 8.6 °C at point IND-2 in December, and the lowest was 1.2 °C in SFH-1 in February (Table 2). The winter pH ranged from 5.5 to 10.2, with the lowest at point IND-2 in January, and the highest at IND-2 in February (Table 2).

**Table 2:** Physicochemical properties of all samples compared in relation to limits for introducing municipal sewage (including storm water, meltwater, and industrial sewage) to water bodies or terrestrial areas (Dz.U.2009.27.169.)

Date	Point	TSS (mg L^−1^)	Temp (°C)	pH	TN	TP	Cl^−^	NO_2_^−^	NO_3_^−^	SO_4_^2−^	Na^2+^	NH_4_^+^	K^+^
					mg L^−1^								
5th December 2010	SFH-2	43	7.2	7.7	1.90	0.16	24	0.09	3	19	19	0.27	2
	IND-2	76	8.6	6.4	1.90	0.21	13	0.06	2	18	9	0.45	2
	IND-1	33	7.4	9.1	0.80	0.39	7	0.06	2	9	6	1.13	2
	BF-1	136	7.6	7.4	2.00	0.90	21	0.07	2	14	14	0.52	3
21st January 2011	SFH-2	40	4.8	9.3	7.70	0.06	246	n.a.	30	91	116	0.25	15
	IND-2	58	7.5	5.5	2.90	0.17	203	0.01	8	250	91	1.08	44
	IND-1	20	6.4	9.3	8.40	0.61	137	0.10	10	82	64	7.28	22
	BF-1	73	6.5	9.4	3.70	0.12	394	0.03	12	136	167	0.45	54
18th February 2011	SFH-2	84	4.3	9.2	5.50	0.44	4510	n.a.	13	114	1947	1.31	20
	IND-2	33	3.9	10.2	2.70	0.39	1243	0.07	4	65	556	1.17	16
	IND-1	54	4.9	8.8	7.50	0.76	4885	n.a.	5	74	2124	4.30	15
	SFH-1	570	1.2	8.8	4.30	0.36	12,086	n.a.	4	76	5445	1.72	7
	BF-1	21	3.9	8.6	5.30	0.78	2487	n.a.	4	92	1127	3.31	34
1st June 2011	SFH-2	192	14.1	8.5	3.20	0.88	6	0.00	1	8	5	0.01	3
	IND-2	41	15.1	8.4	1.90	0.29	7	0.08	2	12	7	0.18	8
	IND-1	93	13.8	8.5	3.20	0.56	8	0.10	2	5	6	0.20	4
	SFH-1	132	14.4	8.3	3.10	0.91	7	0.00	0	3	6	0.01	3
	BF-1	75	14.3	8.5	2.70	0.62	41	0.18	2	15	27	0.37	14
Polish law limits		100^a^	35.0^b^	6.5–9^b^	30.00^b^	2.00^b^	1000^b^	1.00^b^	30^b^	500^b^	800^b^	10.00^b^	80^b^

*n.a.* below the level of detection

^a^Limit for introducing sewage from storm water sewage systems to water bodies

^b^Limit for introducing municipal sewage (including industrial sewage) to water bodies

The highest TSS value, 570 mg L^−1^, was observed at point SFH-1 in February, while the second highest was 136 mg L^−1^, observed at BF-1 in December. The rest of the TSS results ranged from 20 to 84 mg L^−1^, with the lowest being found at IND-1 in January (Table 2).

In terms of nutrient concentrations (Table 2), the TN concentration in winter ranged from 0.8 to 8.4 mg L^−1^: the lowest value was seen in December and the highest in January, both at IND-1. The lowest TP value, 0.06 mg L^−1^, was noted in SFH-2 in January and the highest, 0.9 mg L^−1^, in BF-1 in December. Other forms of nitrogen were within the following ranges in winter: 0.01–0.18 mg NO_2_^−^ L^−1^ (the lowest in IND-2 and the highest in IND-1, both in January); 2–30 mg L^−1^ for NO_3_^−^ (the highest in SFH-2 in January, the lowest in three points in December); and 0.25–7.28 mg NH_4_^+^ L^−1^—the lowest for SFH-2 and the highest for IND-1, both in January. High concentrations of Cl^−^ and Na^+^ were noticed in December for all points (Table 2): from 1243 mg L^−1^ (point IND-2) to 12,086 mg L^−1^ (point SFH-1) for Cl^−^ and from 556 to 5445 mg L^−1^ for Na^+^ for the same respective points. In the rest of the winter samples, the concentration remained below 400 mg Cl^−^ L^−1^ and 170 mg Na^+^ L^−1^, with the lowest values (7 mg Cl^−^ L^−1^ and 6 mg Na^+^ L^−1^) occurring in December at IND-1.

The winter concentration of SO_4_^2−^ was highest in January, with 250 mg L^−1^ for IND-2 and lowest in December with 9 mg L^−1^ for IND-2 (Table 2). Winter K^+^ samples ranged from 44 mg L^−1^, seen at IND-2 in January, and 2 mg L^−1^, found at three points in December. The comparative study in June showed higher water temperatures, ranging from 13.8 to 15.1 °C. TSS concentrations in spring ranged from 41 to 192 mg L^−1^, with only the maximum value being lower than in the winter samples (Table 2). The concentrations of chloride (in the range 6–41 mg L^−1^), sodium (5–27 mg L^−1^), and ammonium (0.01–0.37 mg L^−1^) ions were much lower in June than other samples, their ranges being 6–41 mg L^−1^ for Cl^−^, 5–27 mg L^−1^ for Na^+^, and 0.01–0.37 mg L^−1^ for NH_4_^+^, especially compared to February snowmelt (Table 2). SO_4_^−^, NO_3_^−^, and K^+^ were other ions whose concentration range was found to be lower in samples taken in June. No significant difference was found between the winter and spring samples with regard to the remaining parameters measured in this study: TN, TP, and NO_2_^−^ (Table 2).

### Toxicity tests

The potential toxicity of the collected samples was described using a toxicity classification system proposed by Persoone et al. (2003). Of the 18 analyzed samples, only point SFH-2 in January was found to have no acute hazard (class I) (Table 3). Six samples were classified as having a slight acute hazard (class II), most of them taken in January. The class weight score of samples representing class II was the most variable, ranging from 17 to 80 % (Table 3). An acute hazard (class III) was noted on five occasions, most of which occurred in February. The class weight score was equal or higher than 50 % in almost all samples from class III. Only one studied sample was recorded as a high acute hazard (class IV) and was reported in point SFH-1 in February; the sample also had a high class weight score of 50 % (Table 3). In samples taken in June, the level of toxicity was found to be less diverse than in those taken in winter: four of them demonstrated slight acute hazard (class II) and only one was defined as an acute hazard (class III). The class weight scores for late spring samples were between 17 and 50 % (Table 3).

**Table 3:** Date of sampling for each point. Points where limits specified by Polish law have been exceeded are given (Dz.U.2009.27.169.) as well as acute hazard classes (Persoone et al. 2003)

Date of sampling	Sampling point (with type of catchment)	TSS limit exceeded for introduced storm water sewage	Parameters of introduced municipal sewage exceeded	Acute hazard classes	Class weight score (%)
5th December 2010	SFH-2	−	−	Acute hazard—class III	50
	IND-2	−	pH	Slight acute hazard—class II	80
	IND-1	−	pH	Acute hazard—class III	50
	BF-1	+	−	Slight acute hazard—class II	40
21st January 2011	SFH-2	−	pH, NO_3_^−^	No acute hazard—class I	0
	IND-2	−	pH	Slight acute hazard—class II	20
	IND-1	−	pH	Slight acute hazard—class II	20
	BF-1	−	pH	Slight acute hazard—class II	40
18th February 2011	SFH-2	−	pH, Cl^−^, Na^+^	Acute hazard—class III	50
	IND-2	−	pH, Cl^−^	Slight acute hazard—class II	33
	IND-1	−	Cl^−^, Na^+^	Acute hazard—class III	33
	SFH-1	+	Cl^−^, Na^+^	High acute hazard—class IV	83
	BF-1	−	Cl^−^, Na^+^	Acute hazard—class III	50
1st June 2011	SFH-2	+	−	Slight acute hazard—class II	17
	IND-2	−	−	Slight acute hazard—class II	17
	IND-1	−	−	slight acute hazard—class II	50
	SFH-1	+	−	Acute hazard—class III	50
	BF-1	−	−	Slight acute hazard—class II	33

*+* exceeded limits, − did not exceed limits

Table 4 shows the relationship between the acute hazard classes and the land development classification of the sampled catchments. Samples from SFH catchments were the most variable with regard to acute hazard class. One of them indicated no acute hazard (class I) while another was classified as a high acute hazard (class IV). In addition, two slight acute hazards (class II) and three acute hazards (class III) were observed in other samples from SFH (Table 4). For IND catchments, six samples were classified as slight acute hazard (class II) and two as acute hazard (class III). Samples from the BF catchment were found to have the same acute hazard classes as those from IND, but the number of samples taken from BF was only half that taken from IND (Table 4).

**Table 4:** Acute hazard classes (by Persoone et al. 2003) for each type of land development

	No acute hazard (class I)	Slight acute hazard (class II)	Acute hazard (class III)	High acute hazard (class IV)
SFH	1	2	3	1
IND	0	6	2	0
BF	0	3	1	0

*SFH* single-family houses, *IND* industrial areas, *BF* blocks of flats

**Fig. 3:**
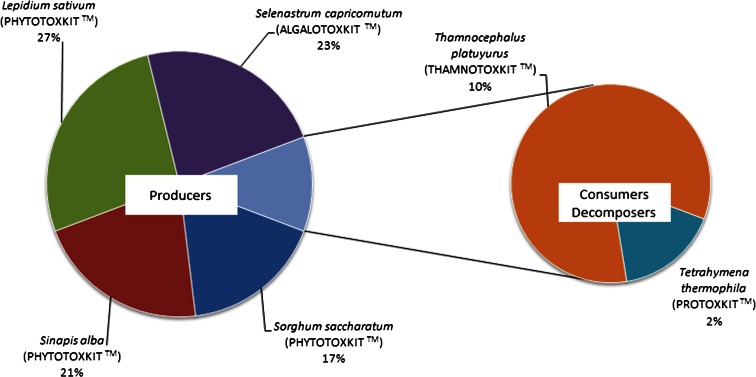
The number of toxic responses described for each applied microbiotest as a percentage of total number of toxic responses

Regarding the toxicity test, it was found that a toxic response, either mortality or growth inhibition, occurred in 17 to 27 % of producers, 10 % of consumers, and 4 % of decomposers. *L. sativum* was the most sensitive test organism in the conducted study, displaying 27 % growth inhibition (Fig. 3).

## Discussion

The biotest results presented herein complement current knowledge regarding the toxic impact of winter storm water and meltwater pollutant fluxes from urban catchments from a variety of drained areas. The toxicity assessment used in this study was found to be a sensitive method for determining runoff quality, with all samples which exceeded the TSS limits defined by Polish Law (Dz.U.2009.27.169.) being classified as slight to acute (class II–IV) hazards (Table 3). This fact, together with previously mentioned TSS ability to absorb toxic contaminants (Gromaire-Mertz et al. 1999; Jartun et al. 2008), means that TSS can be considered as an indicator for the assessment of toxicity evaluation. However, this toxic effect was also observed in 13 other samples, whose TSS level regarded as acceptable according to Polish legislation (Dz.U.2009.27.169.). Therefore, it could be concluded that TSS value did not indicate the presence of an actual toxic threat in most of the analyzed samples.

Further analysis of the physicochemical parameters revealed more samples with pH, Cl^−^, Na^+^, and NO_3_^−^ values, exceeding those set by legislation to be toxic (Tables 2 and 3). However, four other samples were also found to be toxic, even though the maximal values for the TSS levels or physicochemical parameters were not exceeded (Table 3), which may be due to the presence of other toxic compounds in the tested environment that were not analyzed in the present study. This result demonstrates the limitation of taking an approach based on the assessment of an ever longer list of individual pollutants, which carries a risk of not including all possible substances. Either petroleum hydrocarbons or heavy metals may have been responsible for the increased toxicity. A study conducted in Canada (Blaise et al. 2004) demonstrated that oil substances accumulated in wetland sediments carry a potential risk of toxicity to bacteria (*Vibrio fischeri*), algae (*S. capricornutum*), and crustaceans (*Hyalella azteca*). In turn, many heavy metals such as Mn, Fe, Pb, Cu, Cr, or Ni have been demonstrated to exert a toxic effect on organisms such as *V. fischeri*, *S. alba*, *S. capricornutum*, *Daphnia magna*, or *T. platyurus* (Palma et al. 2010; Maisto et al. 2011). Moreover, earlier research on one of the Sokolowka reservoirs in Lodz showed that the concentration of heavy metals (Ni, Cu, Mo, Co, Cd, Pb, Mn, As, and Se) was higher than that present in the reservoir in an agricultural catchment (Urbaniak 2008).

The findings of the present study indicate that the organism most sensitive to the snowmelt and storm water samples was the vascular plant *L. sativum*, a producer (Fig. 3). This could be caused by, inter alia, the sensitivity of the plant to changes in pH. Previous research conducted on *L. sativum* showed that a pH level which was too low or too high could significantly inhibit sprouting and plant development (Oleszczuk 2008). In the present study, in 44 % of all samples, the pH was outside the range 6.5 to 9 accepted by Polish legislation (Table 2).

The highest toxicity period in the present study, with the highest chloride concentrations, was observed in snowmelt February samples (Tables 2 and 3). The highest level of Cl^−^ ions observed in the present study was 12,086 mg L^−1^ at a single family house (SFH-1) point, and this sample was found to be toxic for all tested organisms representing producers (*S. capricornutum*, *S. saccharatum*, *L. sativum*, and *S. alba*) consumers (*T. platyurus),* and decomposers (*T. thermophila*) and was rated as a high acute hazard (class IV) (Table 3). In previous studies, the Cl^−^ ions from road salt have been observed to have a toxic effect on the cyanobacterium *Cylindrospermum* (Chris et al. 2006) and plant *Lemna minor* (Sikorski et al. 2011). Moreover, Waara and Färm (2008) also note that samples taken in January from a highway in Sweden, where the Cl^−^ ion concentration was between 1950 and 2750 mg L^−1^, inhibited the growth of mentioned *L. minor* roots. On the other hand, Waara and Färm (2008) did not observe any toxic effect at other trophic levels, represented by *D. magna* and *T. platyurus* (consumers) and *V. fischeri* (decomposer). In turn, a study by Tixer et al. (2012) on sediments from storm water ponds also reported Cl^−^ ions with a maximum concentration of 4470 mg L^−1^ to have a toxic effect on the consumers *Hexagenia* sp and *H. azteca*. High chloride concentrations in early spring were also toxic for *H. azteca* (Bartlett et al. 2012b) and for the diatom community (Porter-Goff et al. 2013).

The comparison of the winter samples with the June samples revealed that winter was the period with the highest risk of toxicity, which confirmed the findings of Waara and Färm (2008). In the winter period, the most commonly observed score was acute hazard (class III), with a high class weight equal to or higher than 50 % (Table 3).

An important factor influencing the toxicity of storm water and snowmelt samples was the type of land use of the drained areas: the storm water sewage system catchments (Göbel et al. 2007; Tixier et al. 2012; Tang et al. 2013). In the present study, areas developed with industrial units (IND) and blocks of flats (BF) had similar toxicity classes (II and III) with slight acute hazard (class II) (Fig. 3). In areas where single family houses dominated (SFH), a large variety of toxicity classes were reported: from no acute hazard (class I) to high acute hazard (class IV) (Fig. 2). Tang et al. (2013) note high toxicity levels in residential catchments and suggest that illegal point sources of pollution could be responsible.

In turn, the cause of the variation found in the SFH sample toxicity could be connected with varied densities of paved roads and, as a consequence, with the amount of applied chlorides (Kelting et al. 2012). Moreover, the type of roofing material, particularly zinc or copper, also could influence the level of storm water toxicity (Heijerick et al. 2002; Göbel et al. 2007).

Previous research conducted in Lodz was focused on the physicochemical impact of storm water runoff on river water quality and discharge dynamics (Urbaniak 2010; Stolarska et al. 2011; Wagner and Breil 2013). The results show that storm water sewage was highly polluted, especially with chlorides and nutrients. The presented results confirm that the chloride and pH changes may be important physicochemical factors affecting the toxicity of rainwater and snowmelt.

## Conclusion

An ecotoxicological evaluation of urban runoff is needed for a complete assessment of the impact of snowmelt and winter storm water on urban aquatic streams. The physicochemical characterization of storm water and snowmelt samples did not completely reflect their biological activity. Only an evaluation using organisms from different trophic levels revealed a more complete image of the threat posed by urban runoff. The tested organisms (*S. capricornutum*, *S. saccharatum*, *L. sativum*, *S. alba*, *T. platyurus*, and *T. thermophila*) were found to have varied sensitivity to contamination of storm water and snowmelt samples. The producer *L. sativum* demonstrated the highest number of toxicity responses. The lowest sensitivity to toxicity of storm water and snowmelt was presented by the decomposer *T. thermophila*.

The results of the present study indicate that winter might present a greater toxic threat than the rest of the year, especially during snowmelt. The accumulation of road salts and other contaminants in snowcover appears to have the greatest influence in this regard. IND and BF catchments were found to bear the constant threat of toxicity (slight acute hazard, class II, and acute hazard, class III). The SFH catchment was characterized by variable toxicity, with the possibility of occurrence of the highest toxicity levels from slight to high acute hazard: class I to IV. The greater diversity of toxicity in the SFH catchments indicates the need for greater consideration of possible sources of pollution and the introduction of adequate controls to reduce the input of pollutants on these areas.

The biotest results presented herein complement knowledge from previous studies regarding the toxic impact of snowmelt and winter storm water pollutants from different catchment fluxes on urban aquatic streams at different trophic levels. Further research is needed in order to confirm these preliminary conclusions and for further validation of the optimal battery of biotests to be used in assessing urban aquatic ecosystems and the methodology for doing so. Above all, toxicity should be assessed throughout the whole year, and the resulting data should be compared with chemical parameters, especially heavy metal, pesticide, hydrocarbon, and PAH levels, to better understand the impact of anthropogenic activity on the quality of the urban aquatic environment.
